# Potential Pathways Associated With Exaggerated T Follicular Helper Response in Human Autoimmune Diseases

**DOI:** 10.3389/fimmu.2018.01630

**Published:** 2018-07-16

**Authors:** Shu Horiuchi, Hideki Ueno

**Affiliations:** ^1^Department of Microbiology, Icahn School of Medicine at Mount Sinai, New York, NY, United States; ^2^Global Health and Emerging Pathogens Institute, Icahn School of Medicine at Mount Sinai, New York, NY, United States

**Keywords:** T follicular helper, autoimmune diseases, IRF5, *TNFSF4*, STAT4, ustekinumab, IL-23, IL-12

## Abstract

Convincing lines of evidence in both mice and humans show that exaggerated T follicular helper (Tfh) responses is pathogenic in autoimmune diseases. However, the cause of exaggerated Tfh response in humans is still much less clear than in mouse models where genetic factors can be manipulated for *in vivo* testing. Nonetheless, recent advances in our understanding on the mechanisms of human Tfh differentiation and identification of multiple risk loci in genome-wide association studies have revealed several pathways potentially associated with exaggerated Tfh response in human autoimmune diseases. In this review, we will first briefly summarize the differentiation mechanisms of Tfh cells in humans. We describe the features of “Tfh-like” cells recently identified in inflamed tissues of human autoimmune diseases. Then we will discuss how risk loci identified in GWAS are potentially involved in exaggerated Tfh response in human autoimmune diseases.

## Introduction: T Follicular Helper (Tfh) Cells

T follicular helper cells represent a CD4^+^ T cell subset that plays fundamental roles for antibody responses against protein antigens [see also previous reviews for Ref. (1–4)]. Tfh cells are critical for the generation of antibody responses as well as for the establishment of long-lived and high-affinity B cell clones. The most mature Tfh cells reside in germinal centers (GC) in secondary lymphoid organs and provide help specifically to high-affinity B cells that have undergone somatic hypermutations within the GCs. The selected B cells undergo clonal expansion and eventually differentiate into long-lived plasma cells and memory B cells. Less mature Tfh cells also interact with antigen-presenting B cells outside GCs and induce B cell proliferation and differentiation into antibody producing cells. Among them, extrafollicular helper cells, another Tfh-lineage CD4^+^ T cell subset, induce the differentiation of extrafollicular plasma cells. This extrafollicular mechanism mainly contributes to the early generation of specific antibodies after antigen challenge. Extrafollicular helper cells share the phenotype, gene profiles, and the functions with GC Tfh cells.

T follicular helper cells and their precursors are endowed with features to perform these tasks (1–4). After interacting with antigen-presenting dendritic cells (DCs) in T cell-rich area of secondary lymphoid organs, Tfh precursors increase the expression of the chemokine receptor CXCR5 and the G protein-coupled receptor S1PR2, while decreasing the expression of CCR7 and the selectin ligand PSGL1. These changes in the cell surface molecular profile are required for their migration toward and retention within B cell follicles enriched with the chemokine CXCL13. Tfh cells produce large amounts of IL-21, which potently promotes the growth, differentiation, and class-switching of B cells (5, 6). Tfh cells in GCs express high levels of inducible co-stimulator (ICOS), a co-stimulatory molecule crucial for their interactions with B cells. CD40 ligand (CD40L) expressed by Tfh cells provides signals to B cells through CD40 for their differentiation and class-switching. The surface markers commonly used to define human GC Tfh cells and their precursors in lymphoid organs are CXCR5, ICOS, and PD-1. GC Tfh cells express these molecules at high levels, whereas their precursors express at low levels (Table 1). CD127 and CCR7 are expressed by Tfh precursors, but not by GC Tfh cells (7). The combination of CXCR5 and PD-1 is often used to define GC Tfh cells and their precursors also in mice, but ICOS can be broadly expressed by activated CD4^+^ T cells in lymphoid organs (8) and is rarely used to define mouse GC Tfh cells.

**Table 1:** Cell surface makers commonly used to define T follicular helper (Tfh) subsets in lymphoid organs.

	Humans		Mice	
	
	Germinal centers (GCs) Tfh	Pre Tfh	GC Tfh	Pre Tfh
CXCR5	++	+	++	+
PD-1	++	+	++	+
Inducible co-stimulator	++	+	+	+
CD127	−	+	−	+
CCR7	−	− ~ ±	−	− ~ ±

Prolonged Tfh response results in enhanced generation of mature Tfh cells and/or extrafollicular helper cells, and eventually leads to autoimmunity (3, 4). This is convincingly demonstrated in many autoimmune-prone mouse models including Roquin^san/san^ mice, in which CD4^+^ T cells overexpress ICOS and excessive signals through ICOS cause exaggerated Tfh and GC responses (9). Molecular mechanisms for Tfh cell differentiation and the causal link of genetic mutations to exaggerated Tfh responses are relatively well defined in mice (3, 4). However, less is known for human autoimmune diseases. In this review, we will first summarize the differentiation mechanisms of Tfh cells in humans briefly. We will also summarize the features of pathogenic “Tfh-like” cells recently characterized in inflamed tissues of human autoimmune diseases. Last, we will discuss how risk loci identified in GWAS overlap with Tfh-differentiation pathway and how they are potentially associated with exaggerated Tfh response in human autoimmune diseases.

## Differentiation of Tfh Cells in Humans

**Figure 1:**
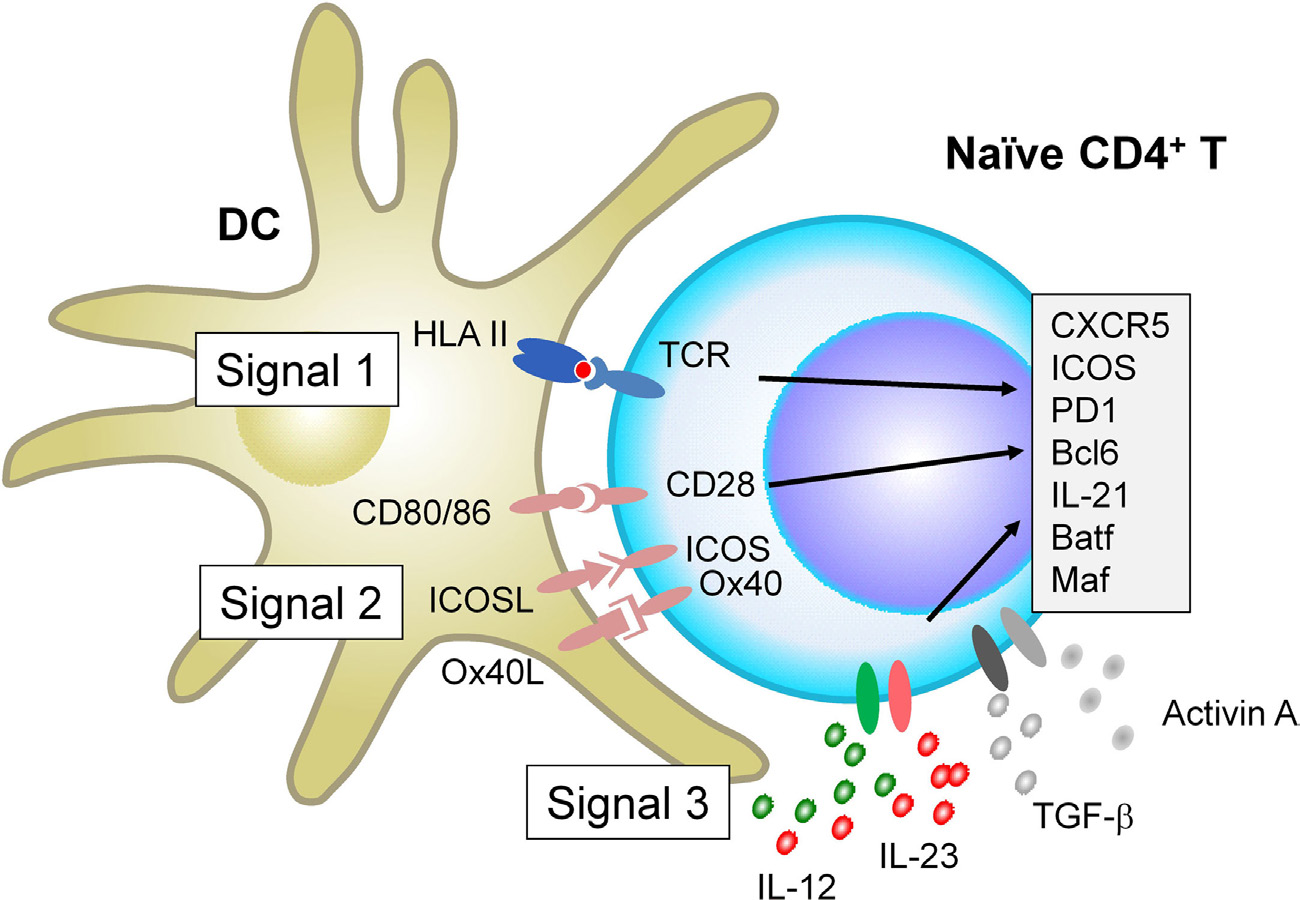
Factors that positively regulate human T follicular helper (Tfh) cell differentiation. When interacting with antigen-presenting dendritic cells (DCs), naïve CD4^+^ T cells receive signals *via* three major receptor families: the T cell receptor (TCR, signal 1), receptors for co-stimulatory molecules (signal 2), and receptors for cytokines (signal 3). For signal 1, evidence in both mice and humans shows that strong TCR signals promote Tfh cell differentiation. For signal 2, in addition to CD28 signals which is essential for optimal T cell activation, signals *via* inducible co-stimulator and Ox40 promote human naïve CD4^+^ T cells to express multiple Tfh molecules. For Signal 3, among inflammatory cytokines that activated DCs produce, IL-12 and IL-23 play dominant roles for human naïve CD4^+^ T cells to express Tfh molecules. The effect of IL-12 and IL-23 is further enhanced by the co-presence of TGF-β family molecules, TGF-β and Activin A. Given that TGF-β and Activin A are often highly expressed in human inflamed tissues, the source of these cytokines might be both from interacting DCs and from microenvironment.

When interacting with antigen-presenting DCs, naïve CD4^+^ T cells receive signals *via* three major receptor families, which ultimately determine the fate of T cell differentiation: the T cell receptor (TCR, signal 1), receptors for co-stimulatory molecules (signal 2), and receptors for cytokines (signal 3). Recent studies show that each signal provides parameters that negatively and positively affect Tfh differentiation in humans and mice (Figure 1).

### Signal 1: TCR

Studies in mice demonstrated that strong TCR signals are required for the differentiation of fully mature Tfh cells (8, 10). Consistent with this, stimulation with stronger TCR signals *in vitro* induces human naïve CD4^+^ T cells to express higher levels of multiple Tfh molecules, including CXCR5, Bcl6, IL-21, and Ox40 (11). As demonstrated in experimental mouse models (8, 10), it is possible that human Tfh cell clones display relatively higher TCR affinity than non-Tfh cell clones, yet this remains to be tested.

### Signal 2: Co-Stimulatory Molecules

Inducible co-stimulator is critically involved in Tfh cell biology at multiple levels, including the differentiation program at early stages (12, 13), their migration into B cell follicles (14), and the functions when interacting with B cells (15, 16). Patients with ICOS deficiency display severely impaired Tfh response accompanied by severely impaired memory B cell formation, indicating the essential role of ICOS in humans (17). Ox40 is another important co-stimulatory molecule promoting human Tfh cell differentiation. Ox40 signals together with TCR and CD28 signals promote human naïve and memory CD4^+^ T cells to express multiple Tfh molecules, including CXCR5, ICOS, PD-1, and Bcl6 (11). The direct contribution of Ox40 signals to Tfh cell differentiation was also recently demonstrated in mice with vaccinia viral infection (18). Unlike ICOS deficiency, however, loss-of-function (*LOF*) mutations in human Ox40 do not affect Tfh and antibody responses (19), suggesting that deficiency of Ox40 signals can be compensated by other mechanisms for Tfh differentiation. Instead, excessive Ox40 signals drive human CD4^+^ T cell differentiation toward Tfh cells and contribute to autoimmunity by increasing Tfh responses (11).

### Signal 3: Cytokines

In humans, IL-12 produced by DCs is the major DC-derived cytokine driving human naïve CD4^+^ T cells to become Tfh-like cells (20–24). This is in contrast to mouse CD4^+^ T cells which acquire multiple features of Tfh cells in response to IL-6 and IL-21 (25, 26). Children who lack the expression of functional IL-12 receptor β1 chain, a common receptor for IL-12 and IL-23, display less Tfh cells and memory B cells in blood circulation and impaired GC formation in lymph nodes, providing *in vivo* evidence of the significance of this pathway for intact Tfh response in humans (21). Another important set of cytokines for human Tfh cell differentiation is TGF-β family cytokines TGF-β and Activin A, which activate the Smad signaling pathways including Smad2 and Smad3. Although only marginally effective by themselves, TGF-β and Activin A co-operate with IL-12 and IL-23 to promote human naïve CD4^+^ T cell differentiation toward the Tfh lineage (23, 27). TGF-β signals render STAT4 and STAT3 (activated by IL-12 and IL-23) to promote human naïve CD4^+^ T cells to acquire Tfh gene signature, while suppressing Th2 and regulatory T cell gene signatures (23). Furthermore, both TGF-β and Activin A also induce human CD4^+^ T cells to produce CXCL13 (27, 28), the major chemokine that human mature Tfh cells produce (7). TGF-β and Activin A are often strongly expressed in inflammatory sites, such as synovial fluid in rheumatoid arthritis (RA) (29, 30). Of note, neither TGF-β nor Activin A, even in the presence of Tfh-promoting cytokines, such as IL-6 and IL-21, induces Tfh molecules in mouse CD4^+^ T cells, and, therefore, this pathway is not shared in mice (23, 27).

Several cytokines are known to inhibit human Tfh cell differentiation. Type I (IFN-α, β, and ω) and type III (IFN-λ1 and λ2) interferons are potent inhibitors of Tfh cell differentiation in humans, and strongly diminish the expression of Tfh markers and gene signature by human naïve CD4^+^ T cells (23). This suggests that exaggerated Tfh cell responses in human autoimmune diseases with dominant IFN signature, such as systemic lupus erythematosus (SLE), is not mediated by the direct effect of type I IFNs on T cells, but by an indirect effect on APCs. Type I IFNs promote human DCs to produce Tfh-promoting cytokines, such as IL-12, IL-23, and IL-6 (31). Similarly, mouse studies demonstrated that type I IFN signals act as an adjuvant for antibody responses by promoting DCs to produce IL-6 (32, 33).

In mice, the IL-2–STAT5–Blimp1 axis inhibits Tfh cell differentiation at multiple differentiation stages (34, 35), and deprivation of IL-2 signals in the microenvironment is important for Tfh cell differentiation (36). There is evidence that IL-2 suppresses also human Tfh cell differentiation. IL-2 inhibition by a neutralizing Ab increased CXCR5 expression by human naïve CD4^+^ T cells cultured in Tfh-promoting culture conditions *in vitro* (27). Furthermore, treatment with low dose IL-2 decreased CXCR5^+^PD-1^+^CCR7^lo^ cTfh cells in SLE accompanied with marked decrease of disease activity (37). It is of note, however, that it is unclear whether decrease of disease activity was dependent on decreased Tfh activity and whether decreased Tfh activity was the primary effect of IL-2 on Tfh cells. Current evidence highly suggests that the major target of low dose IL-2 treatment was Tregs. Low dose IL-2 treatment selectively induced STAT5 activation in Tregs but not conventional CD4^+^ T cells *in vitro* (38). In chronic GVHD patients, low dose IL-2 expanded circulating Treg frequency and their suppressive functions (38, 39). Therefore, it is possible that decrease of Tfh activity was secondary to increased activity of Tregs.

## “Tfh-Like” Cells in Inflamed Tissues of Human Autoimmune Diseases

Recent studies have identified “Tfh-like” cells involved in autoantibody production in inflamed tissues of human autoimmune diseases. These T cells share properties with Tfh cells only partly. Characterization of CD4^+^ T cells in inflamed synovial tissues in RA patients revealed a massive expansion of CXCR5^−^PD-1^+^ CD4^+^ T cells. While these cells expressed many human Tfh markers, including IL-21, CXCL13, ICOS, CD84, TIGIT, and c-Maf, and were able to induce memory B cells to produce IgG *in vitro*, they lacked the expression of CXCR5 and Bcl6. CXCR5^−^PD-1^+^ cells instead expressed Blimp-1 and the chemokine receptors CCR2, CCR5, and CX3CR1 (40). Analysis of inflamed salivary glands in Sjogren’s syndrome showed an increase of CXCR5^−^ ICOS^+^ CCR9^+^ CD4^+^ T cells. CCR9^+^ CD4^+^ T cells isolated from blood samples of patients produced IL-21 upon activation and were capable of inducing memory B cells to produce IgG (41). These studies suggest that the precise nature of B helper T cells associated with autoantibody production in inflamed tissues might differ among diseases, likely reflecting the differences in microenvironment. The chemokine receptors and integrins expressed by these “Tfh-like” cells might be critically involved for their migration to and retention in inflammatory tissues. It will be important to clarify how the developmental mechanisms and the functional regulations of “Tfh-like” cells are different from Tfh cells in lymphoid organs.

## Transcription Factor Network in Tfh Cell Differentiation

CD4^+^ T cell differentiation is controlled by transcription factors which govern the expression of a set of target genes. Recent studies in mice identified a number of transcription factors and pathways that affect Tfh cell differentiation. Transcription factors positively regulate Tfh cell differentiation include Bcl6, Ascl2, Batf, cMaf, IRF4, STAT3, STAT1, STAT4, NOTCH1, NOTCH2, Tcf1, and Lef1 (42–48). Among these, Bcl6 is specifically required for the differentiation of Tfh cells, but not other CD4^+^ T cell subsets (7, 44, 49–51). The transcription factor Blimp-1, an antagonist of Bcl6, inhibits Tfh cell differentiation (51). Genome-wide analysis of Bcl6-binding sites by using ChIP-Seq demonstrated that Bcl6 binds to genes associated with the differentiation of other CD4^+^ T cell lineages, including Th1, Th2, Th17, and Tregs, and genes that negatively affect CD4^+^ T cell localization in GCs (52). Thus, Bcl6 primarily acts as a repressor to inhibit the differentiation toward other CD4^+^ T cell lineages and to diminish the molecules that exclude CD4^+^ T cells from GCs (49–52). Interestingly, many genes repressed by Bcl6 contained AP1 and STAT motifs but lacked Bcl6 motif, suggesting that AP1 and STAT recruit Bcl6 to the target sites and repress the target genes (52).

## Risk Loci Identified in GWAS of Autoimmune Diseases and Their Potential Association with Exaggerated Tfh Response

**Figure 2:**
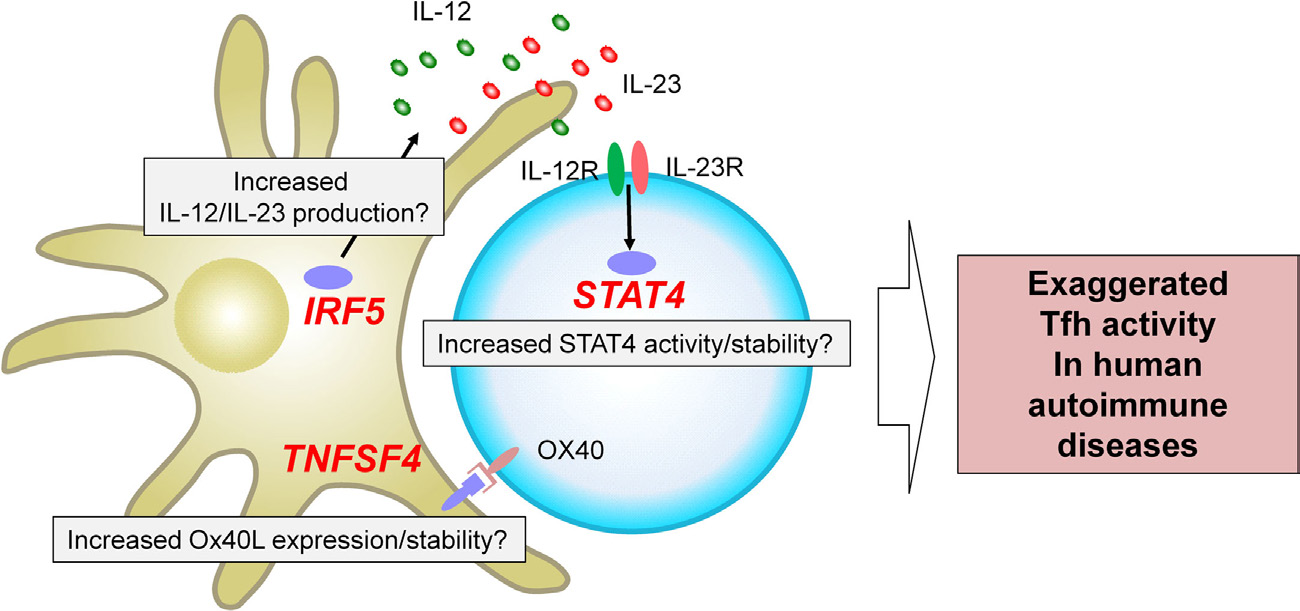
Three risk loci potentially associated with exaggerated T follicular helper (Tfh) response in human autoantibody-mediated autoimmune diseases. GWAS in autoimmune diseases have identified multiple loci associated with disease risk. *STAT4, IRF5*, and *TNFSF4* have been identified and validated in multiple studies on autoantibody mediated autoimmune diseases as risk loci with a strong disease association at the genome-wide *p* value of at least 5 × 10^−8^. These risk loci might contribute to the exaggerated Tfh response in human autoimmune diseases. For example, *IRF5* risk loci might be associated with an increased production of Tfh-promoting cytokines, including IL-12, IL-23, and IL-6. *TNFSF4* risk loci might contribute to increased Ox40 signals that promote the expression of Tfh molecules by human CD4^+^ T cells. *STAT4* risk loci might affect the activity and/or stability of STAT4 and might enhance the signals of IL-12 and IL-23.

The genome-wide association studies (53) are an experimental design used to detect associations between genetic variants and traits including diseases in samples from populations (54). GWAS in autoimmune diseases have successfully identified loci associated with an increase or a decrease of risk (53–55). A fraction of the identified risk loci is located within or in the proximity of genes encoding transcription factors and cell surface molecules expressed by T cells and/or antigen-presenting cells. How risk haplotypes are associated with their dysregulated function and linked to the immunological consequences largely remains to be established. Nonetheless, several key risk loci are located in the proximity of genes that regulate Tfh differentiation in humans, and thus might contribute to the exaggerated Tfh response in human autoimmune diseases. Here we focus on three genes, *STAT4, IRF5*, and *TNFSF4*, which are identified and validated as risk loci in GWAS in multiple autoantibody mediated autoimmune diseases with a strong disease association at the genome-wide *p* value of at least 5 × 10^−8^ (Figure 2).

## STAT4

STAT4 is among the top risk loci (other than HLA genes) identified in GWAS on SLE, RA, and Sjogren’s syndrome (56). STAT4 is dominantly activated by IL-12 and to a lesser extent by IL-23 and type I IFNs (57). As discussed above, the IL-12–STAT4 pathway strongly promotes Tfh cell differentiation in humans. Human naïve CD4^+^ T cells primed in the presence of IL-12 highly upregulate expression of multiple Tfh molecules (21, 58), which are inhibited by decreasing STAT4 expression by specific siRNA transfection (22, 23). STAT4 in CD4^+^ T cells in the proximity of GCs in inflamed human tonsils is strongly phosphorylated, indicating that the IL-12–STAT4 pathway is active *in situ* (23). Previous studies showed that DCs and macrophages in inflamed lymphoid organs and tissues of autoimmune diseases express IL-12 and IL-23 (31). It is plausible that STAT4 allele variants [such as rs11889341-T in SLE (59)] might be directly involved in the exaggeration of Tfh response, for example, by increasing their signaling efficacy and/or stability of STAT4 in CD4^+^ T cells stimulated by IL-12 and/or IL-23. Of note, the IL-12–STAT4 pathway contributes to the Tfh differentiation also in mice, and STAT4 directly binds to multiple Tfh genes in IL-12-stimulated mouse CD4^+^ T cells, including *Il21, Cxcr5, Pdcd1* (encoding PD-1), and *Icos* (60, 61).

A recent report showed that ustekinumab, a human recombinant antibody against IL-12p40 (a component of IL-12 and IL-23), was effective for active SLE in a phase II trial (presented in ACR 2017 meeting). Although this encouraging outcome remains to be confirmed, it is of great interest to determine whether the treatment with ustekinumab reduced Tfh responses, and more importantly whether the reduction of Tfh response correlated with the improvement of clinical parameters. Alternatively, treatment with ustekinumab might target other immune cells including Th1 and Th17 cells, which are also proposed to play pathogenic roles in SLE (62, 63). Determining the mode of action of ustekinumab will provide critical insights into the type of pathogenic CD4^+^ T cells and the pathways in SLE.

Other STAT molecules STAT3 and STAT1 also positively regulate Tfh cell differentiation in mice (4). STAT3 is also critical for human Tfh cell differentiation, as patients with STAT3 *LOF* mutations as well as patients with IL-21 receptor deficiency display significantly less cTfh cells (64). Furthermore, CD4^+^ T cells from patients with STAT3 *LOF* mutations and IL-21 receptor deficiency produce substantially less IL-21 upon IL-12 stimulation (24), suggesting that the IL-21–STAT3 axis is critical for enhancement of IL-21 expression. Interestingly, despite less IL-21 production, STAT3 *LOF* human CD4^+^ T cells stimulated with IL-12 express normal levels of CXCR5, ICOS, and Bcl-6 (26). Thus, STAT3 and STAT4 might differently contribute to the expression of each Tfh molecule in human CD4^+^ T cells, and IL-21 expression might be more dependent on the IL-21–STAT3 axis than the expression of CXCR5, ICOS, and Bcl-6. In this line, it is likely that decreased cTfh cells in STAT3 *LOF* are also due to severely impaired B cell response. STAT3 *LOF* display significantly less memory B cells (65), and the differentiation of STAT3 *LOF* B cells into plasmablast is severely impaired due to failure to respond to IL-6, IL-10, or IL-21 (65). Notably, unlike STAT4, STAT3 has been identified as risk loci in GWAS in autoinflammatory diseases, such as multiple sclerosis, Crohn’s disease, and ulcerative colitis, which are less dependent on autoantibodies (53). STAT1 signals seems to play less significant roles in Tfh cell differentiation in humans than in mice, as patients with both STAT1 *LOF* and gain-of-function (*GOF*) mutations display normal frequency of cTfh cells (24, 58, 64). No GWAS on autoimmune diseases have identified STAT1 as a risk allele. These observations suggest that genetic variants of STAT1 and STAT3 do not contribute much to pathogenic Tfh and GC responses in autoimmune diseases. However, this notion does not preclude the involvement of STAT3 mutations in human autoimmune diseases. Patients with STAT3 *GOF* mutations show massive multiorgan autoimmunity, autoimmune cytopenia, lymphoproliferation, and immunodeficiency, likely due to impaired Treg differentiation and functions (66–68). Whether STAT3 *GOF* increases Tfh response or not is currently unclear. However, many patients display hypogammaglobulinemia (68), and thus that autoimmunity in STAT3 *GOF* seems to be caused by dysregulation of antibody response rather than a general increase of Tfh response.

## IRF5

IRF5 is another transcription factor whose haplotype is highly associated with an increased risk of SLE, RA, and Sjogren’s syndrome (53). IRF5 is expressed by a broad range of APCs, including monocytes, macrophages, DCs, plasmacytoid DCs, and B cells, and is critically involved in the production of inflammatory cytokines, including IL-12, IL-23, IL-6, and type I IFN (69–71). These cytokines are enriched in inflamed tissues in autoimmune diseases, and such inflammatory cytokine milieu causes a positive-feedback loop for the expression of IL-12 and IL-23 by promoting the generating M1 macrophages that highly express IRF5 (69). Thus, it is possible that IRF5 risk haplotype is linked to the enhanced production of Tfh-promoting cytokines in subjects with autoimmune diseases traits. IRF5 is required in normal human B cell proliferation and differentiation into plasmablasts (72), and therefore, IRF5 risk allele may also intrinsically affect B cells. The importance of IRF5 for lupus pathogenesis was also demonstrated by the attenuation of the disease in mouse models deficient of IRF5, which was accompanied with decreased activated CD4^+^ T cells and autoantibodies (73, 74).

## *TNFSF4* 

*TNFSF4* encodes Ox40L, a TNF ligand family molecule that delivers signals promoting human Tfh cell differentiation (11) as described earlier. *TNFSF4* has been identified as another prominent risk allele in SLE and RA (53). In active SLE, blood CD14^+^ monocytes and tissue CD11c^+^ myeloid cells upregulate the expression of Ox40L (11). *TNFSF4* risk allele might enhance signals *via* Ox40 and contribute to enhanced Tfh responses. The importance of the Ox40–Ox40L axis for antibody response (75–77) and autoimmune disease was also demonstrated in studies with mouse models (18, 78, 79). *TNFSF4* is also found as a risk allele in GWAS on allergies, such as asthma, hay fever, and eczema; an observation consistent with the importance of Ox40–Ox40L pathway for Th2 response (80).

In contrast, it is somewhat surprising that ICOS, another key co-stimulatory molecule for Tfh cell differentiation in humans and mice, and its ligand ICOS-ligand (encoded by *ICOSLG*) are not among the risk loci identified in GWAS on autoantibody mediated autoimmune diseases. *ICOSLG* was instead found to be highly associated with Crohn’s disease and ulcerative colitis (53). Thus, while ICOS and ICOS-ligand are involved in pathogenic Tfh response, as demonstrated in Roquin^san/san^ mice, the haplotype *ICOS* and *ICOSLG* themselves *per se* do not seem to contribute to pathogenic Tfh responses.

## Concluding Remarks

Although the mode of actions remains to be established, a positive outcome of ustekinumab in active SLE patients provides a new proof of principle that T cells can be a therapeutic target for autoantibody-mediated autoimmune diseases. Another evidence came from a clinical trial performed some 15 years ago with anti-CD40L (BG9588) on lupus nephritis. Although the trial was prematurely terminated due to the development of thrombotic events and associated fatality in some patients, the early results were encouraging and the treatment reduced serum anti-dsDNA titers, increased serum complement levels, and reduced nephritis score (81). Thus, targeting B helper T cells can be beneficial for treatment of autoimmune diseases. It will be important to define the differentiation mechanisms and the functions of Tfh and Tfh-like cells that directly contribute to the production of autoantibodies in lymphoid organs as well as inflamed tissues. Determining how risk loci in autoimmune diseases are associated with the molecular mechanisms for exaggerated Tfh responses is also of great importance. These studies will enhance our understanding in human autoimmune diseases and also might provide novel therapeutic targets.

## Author Contributions

HU and SH, have made a substantial, direct, and intellectual contribution to the work and approved it for publication.

## Conflict of Interest Statement

The authors declare that the research was conducted in the absence of any commercial or financial relationships that could be construed as a potential conflict of interest.
